# Early Degeneration of Both Dopaminergic and Serotonergic Axons – A Common Mechanism in Parkinson’s Disease

**DOI:** 10.3389/fncel.2016.00293

**Published:** 2016-12-22

**Authors:** Janina Grosch, Jürgen Winkler, Zacharias Kohl

**Affiliations:** Department of Molecular Neurology, Friedrich-Alexander University Erlangen-NürnbergErlangen, Germany

**Keywords:** Parkinson’s disease, axon degeneration, dying back, synaptic loss, dopamine, 5-HT

## Abstract

Motor symptoms in Parkinson’s disease (PD) are tightly linked to the degeneration of substantia nigra dopaminergic neurons and their projections into the striatum. Moreover, a broad range of non-motor symptoms like anxiety and depression frequently occur in PD, most likely related to the loss of serotonergic neurons and their projections into corresponding target regions. Strikingly, nigral dopaminergic neurons and raphe serotonergic neurons are severely affected in PD showing characteristic hallmarks of PD neuropathology, in particular alpha-synuclein containing Lewy bodies and Lewy neurites. So far, the initial events underlying neurodegenerative processes in PD are not well understood. Several observations, however, indicate that neurites and synapses of diseased neurons may be the first subcellular compartments compromised by alpha-synuclein associated pathology. In particular axonal pathology and deficits in axonal transport may be leading to the onset of synucleinopathies such as PD. This review will highlight current findings derived from imaging and neuropathological studies in PD patients, as well as cellular and animal PD models, which define the initial underlying structural and molecular events within dopaminergic and serotonergic circuits leading to the ‘dying back’ degeneration of axonal projections in PD.

## Introduction

Parkinson’s disease (PD) represents the second most common neurodegenerative disorder, affecting up to ten million people worldwide (de Lau and Breteler, 2006), with a predicted increase of more than twofold by 2030 (Dorsey et al., 2007). PD patients show progressive motor symptoms such as bradykinesia, rigidity, and resting tremor. Moreover, PD is accompanied by a broad range of non-motor symptoms (NMS), including cognitive deficits, autonomic dysfunctions, and mood disorders (Marras and Chaudhuri, 2016). While the etiology of PD still remains unclear, the aggregation of the presynaptic protein alpha-synuclein, either within Lewy bodies or Lewy neurites of susceptible neurons is of ultimate diagnostic value (Dickson et al., 2009). The other major neuropathological hallmark of PD is the degeneration and subsequent loss of dopaminergic neurons in the substantia nigra (SN) leading to prototypic motor deficits (Greffard et al., 2006). Interestingly, Lewy pathology was also described in numerous other neuronal subpopulations in distinct brain regions, most likely occurring even prior to dopaminergic SN neurons, e.g., in the medulla oblongata, olfactory bulb, and pontine tegmentum (Braak et al., 2003). These findings are paralleled by deficits of several neurotransmitter systems including serotonergic, noradrenergic, cholinergic, GABAergic, and glutamatergic signaling (reviewed in Brichta et al., 2013). PD pathology in these non-dopaminergic systems, in particular serotonergic neurotransmission, may be related to NMS like anhedonia, anxiety and depression, affecting up to 50% of PD patients and having a strong impact on patients’ quality of life (Gallagher and Schrag, 2012; Duncan et al., 2014; Bugalho et al., 2016).

Several studies imply that dysfunction of different neurotransmitter systems occur even prior to the presence of Lewy pathology and the consequent neuronal loss [e.g., reviewed in (Burke and O’Malley, 2013)]. This led to the overall hypothesis, that the main pathophysiological process is related to a “dying back” of axons prior to the loss of neuronal perikarya (Hornykiewicz, 1998; Cheng et al., 2010). This review will discuss recent findings of clinical and neuropathological studies, as well as molecular mechanisms from cellular and animal models supporting the concept of an early axonal pathology in PD.

## Loss of Dopaminergic Neurons and Their Projections in PD

The involvement of the nigrostriatal dopaminergic system in PD was noted as early as neuropathological studies detected the loss of melanin-containing dopaminergic neurons in the SN of affected patients (Pakkenberg and Brody, 1965; Hirsch et al., 1988). The extent of neuronal loss in the SN by the time of clinical diagnosis was subject of several subsequent studies: While many reviews repeatedly state that first motor symptoms appear after 50% of SN dopaminergic neurons are lost (Lang and Lozano, 1998; Nandhagopal et al., 2008), comprehensive clinico-neuropathological studies used regression analysis to more precisely determine the loss of dopaminergic neurons at the time of symptom onset. Fearnley and Lees (1991) estimated the percentage of lost pigmented SN neurons at symptom onset by 31%. Subsequent stereological analyses provided similar results extrapolating the number of remaining pigmented SN neurons to the time of motor symptom onset to about 70% (Ma et al., 1997; Greffard et al., 2006). In summary, neuropathological-clinical correlations suggest that initial motor signs in PD occur as early as around 30% of total SN neurons are lost.

In contrast, the loss of dopaminergic striatal nerve terminals at motor symptoms onset is rather difficult to determine: An early neurochemical study focused on caudate dopamine levels in *post-mortem* tissue in two different cohorts (about 60 years vs. 73 years of age), and allowed an extrapolation to the time of symptom onset ranging from 68 to 82% of dopamine reduction (Riederer and Wuketich, 1976). To circumvent the concerns about *post-mortem* delay, another study utilized measurement of vesicular monoamine transporter (VMAT2) binding with tritiated alpha-dihydrotetrabenazine ([^3^H]TBZOH) in *post-mortem* caudate from PD patients with different disease duration (Scherman et al., 1989). Here, the regression analysis indicated a loss of 49% of binding sites by the time of motor symptom onset. In general, the loss of dopamine terminals is higher in the putamen compared to the caudate (Kish et al., 1988), allowing the assumption that the loss of binding in the putamen ranges even higher than 50% by the time of onset of motor symptoms. Recently, Kordower et al. (2013) described in detail the integrity of nigrostriatal dopaminergic connectivity using tyrosine hydroxylase (TH) and dopamine transporter (DAT) as markers for dopaminergic function: In PD, dopaminergic fiber density in the dorsal putamen was rapidly and severely decreased leading to a virtually complete neuritic loss at 4 years after the clinical diagnosis compared to controls. In contrast, TH^+^ SN neurons were less severely affected from the earliest time points on, with a minor loss over time, resulting in a residual population of dopaminergic neurons even decades after diagnosis (Kordower et al., 2013). In another study, this group detected an early loss of kinesin protein (anterograde transport motor protein) in the putamen of PD patients (H&Y stage 1 and 2), representing axonal loss, while reductions of typical cytoplasmic proteins like dynein light chain Tctex 3 as well as TH protein levels in the SN were only observed at later PD stages (Chu et al., 2012). Taken together, these observations support the hypothesis of an early axonal degeneration involving transport deficits in PD.

## Impaired Nigrostriatal Dopaminergic Projections *In Vivo*

While there are concerns about the significance of neuro-pathological and -chemical measures due to *post-mortem* delay, several *in vivo* studies investigated axonal or synaptic loss by using radioligand imaging (reviewed recently by Politis, 2014), in particular measuring DAT and VMAT2 (**Table 1**). Again, particularly those studies performing regression analysis to the onset of motor symptoms are relevant: the proportion of lost striatal and putaminal dopaminergic terminals was estimated up to 56% (Schwartz et al., 2004). Using [^123^I]beta-CIT SPECT to label presynaptic DAT an early study described a loss of dopaminergic terminals contralateral to the unaffected side by 39–51% in the striatum and 51–64% in the putamen (Tissingh et al., 1998). In a larger study using two different PET markers, for DAT and VMAT2, the loss of putaminal dopaminergic innervation was between 51 and 71% for both tracers (Lee et al., 2000). More recent work using these established PET markers suggests that younger PD patients are able to compensate over several years the progressive dysfunction of the dopaminergic system before the first motor symptoms are recognized (de la Fuente-Fernandez et al., 2011).

**Table 1:** Parkinson’s disease (PD) imaging studies of dopaminergic and serotonergic deficits.

Modality	Method	Ligand	*N*	Region	Marker loss (%)	Type of analysis	Reference
**Dopamine imaging**					
DAT binding	SPECT	[^123^I]- IPT	6	Striatum	43	Regression to time of symptom onset	Schwartz et al., 2004
				Putamen	56		
	SPECT	[^123^I]- β-CIT	8 (H&Y I)	Striatum	39–51	H&Y I (ipsi- vs. contralateral)	Tissingh et al., 1998
				Putamen	51–64		
	PET	[^11^C]- MP	13 (H&Y I)	Putamen	56–71	H&Y I (ipsi- vs. contralateral)	Lee et al., 2000
VMAT2 binding	PET	[^11^C]- DTBZ	13 (H&Y I)	Putamen	51–62	H&Y I (ipsi- vs. contralateral)	Lee et al., 2000
	PET	[^11^C]- DTBZ	78 (H&Y I-II)	Putamen	71 (younger) 34 (older)	Regression to time of symptom onset; younger vs. older age of onset	de la Fuente-Fernandez et al., 2011
**Serotonin imaging**					
SERT binding	PET	[^11^C]McN5652	13	Caudate	50	H&Y I-IV vs. controls	Kerenyi et al., 2003
				Putamen	35		
	PET	[^11^C]- DASB	9	Caudate	30	H&Y II-III vs. controls	Guttman et al., 2007
				Putamen	26		
				Midbrain	29		
				Orbitofrontal cortex	22		
	PET	[^11^C]- DASB	5	Forebrain regions	40–50	H&Y I-II,5 vs. controls	Albin et al., 2008
				Caudal brain stem regions	∼20		
	PET	[^11^C]- DASB	30	Caudate	28	H&Y I-II vs. controls	Politis et al., 2010
				Thalamus	17		
				Anterior cingulate cortex	32		
				+ Putamen	33	H&Y II-III vs. controls	
				+ Prefrontal cortex	40		
				+ Raphe nuclei	19/22	H&Y III-IV vs. controls	
				+ Amygdala	25		
	PET	[^11^C]- DASB	30	Ventral striatum Anterior cingulate cortex Caudate (R) Orbitofrontal cortex (R)	Significant loss	Apathetic vs. non-apathetic drug naïve early PD	Maillet et al., 2016

*IPT, N-(3-iodopropen-2-yl)-2beta-carboxymethoxy-3beta-(4-chlorophenyl)-tropane; β-CIT, beta-carbomethoxy-3 beta-(4-iodophenyltropane); MP, methylphenidate; DTBZ, dihydrotetrabenazine; McN5652, (l,2,3,5,6,10βhexahydro6[4(methylthio) phenyl] pyrrolo[2,1-a]isoquinoline); DASB, 3-amino-4-(2-dimethylaminomethyl-phenylsufanyl-)benzonitrile.*

At present, neuro-pathological and -imaging findings suggest that at the onset of motor symptoms in PD the loss of striatal or putaminal dopaminergic projections largely exceeds that of dopaminergic SN neurons, again supporting the hypothesis of a “dying back” of axons to their dopaminergic perikarya in PD (Cheng et al., 2010; Chu et al., 2012; Burke and O’Malley, 2013).

## Loss of Serotonergic Neurons AND Their Projections in PD

Serotonergic neurotransmission is widely distributed in the brain and mediated by serotonergic neurons in the raphe nuclei (RN). Clusters of rostral RN neurons mainly project to the forebrain innervating basal ganglia, amygdala, hippocampus, hypothalamus, and several cortical regions (Parent et al., 2011; Wallman et al., 2011). Important functions are linked to the serotonergic system including motor function, as well as cognition and mood: Dysfunction of serotonin (5-hydroxytryptamine, 5-HT) neurotransmission contributes to resting tremor and levodopa-induced dyskinesias (Doder et al., 2003; Rylander et al., 2010; Politis et al., 2014) as well as to typical NMS of PD, in particular apathy, anxiety, anhedonia, and depression (Pavese et al., 2010; Ballanger et al., 2012).

Neuropathological analyses demonstrated an early involvement of serotonergic neurons in PD (Halliday et al., 1990; Paulus and Jellinger, 1991), associated with the presence of Lewy pathology within the RN at an early disease stage (Braak et al., 2003; Seidel et al., 2015). In detail, work by Halliday et al. (1990) described a loss of serotonergic neurons in the median RN of PD patients by 56%. Intriguingly, serotonergic cell loss was correlated with depression in PD, revealing an increased cell loss in the RN of depressed PD patients (Paulus and Jellinger, 1991). Typical NMS, possibly related to the serotonergic system like anxiety or depression, are often present before the onset of motor symptoms (Weintraub et al., 2015). Moreover, serotonin depletion was observed in several target regions of the RN including basal ganglia, hypothalamus, hippocampus, and prefrontal cortex (Fahn et al., 1971; Shannak et al., 1994). In addition, levels of serotonin transporter (SERT) immunoreactivity, protein levels of tryptophan hydroxylase, 5-HT and its metabolites were reduced in the caudate nucleus compared to the putamen in *post-mortem* tissue from PD patients (Kish et al., 2008).

## *In Vivo* Studies Addressing the Serotonergic System in PD

Specific radioligands for the serotonergic system allowed new insights in PD related alterations *in vivo* (**Table 1**): Besides selective radiotracers for 5-HT receptors, particularly the highly selective detection of SERT using [^11^C]-DASB PET enabled tracing of serotonergic terminals in the brain. Early studies detected a reduced SERT binding in the caudate nucleus and the putamen of PD patients (Kerenyi et al., 2003; Guttman et al., 2007). A follow-up study stratified for disease duration and without history of depression, described in detail an early loss of serotonergic terminals in the caudate nucleus, thalamus, hypothalamus, and anterior cingulum, followed by further deficits in putamen, insula, posterior cingulum, and prefrontal cortex in PD (Politis et al., 2010). Interestingly, the loss of SERT binding in the caudal and rostral RN occurred in advanced disease stages only, which points to an earlier affection of serotonergic projections compared to serotonergic neurons. This notion was supported by another [^11^C]-DASB-PET study showing reduced forebrain, but preserved brain stem SERT binding in PD patients (Albin et al., 2008). Interestingly, a recent PET study in PD patients without signs of apathy mainly showed dopaminergic denervation, while serotonergic innervation remained preserved. In contrast, PD patients with apathy exhibited additional serotonergic loss within the right caudate, insula, orbitofrontal cortex, and cingulum without prominent dopaminergic deficits (Maillet et al., 2016). Despite these new insights into the dysfunction of the serotonergic system in PD, a temporal dissociation of the loss of serotonergic terminals in the forebrain and the number of RN neurons has not been well established, although these imaging studies point to an early degeneration of serotonergic nerve terminals.

## Axonal Degeneration in Murine PD Models

**FIGURE 1:**
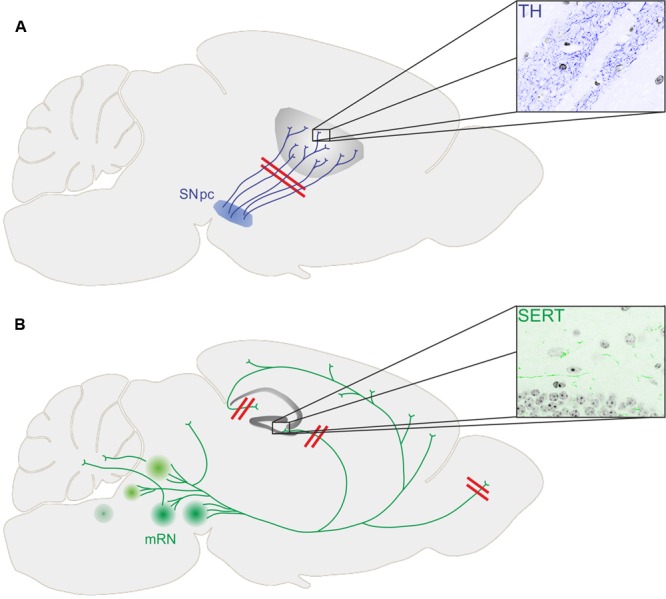
**(A)** Loss of dopaminergic terminals (stained for tyrosine hydroxylase, TH) in the striatum precedes loss of substantia nigra pars compacta (SNpc) dopaminergic neurons in transgenic mouse models of PD. **(B)** While the number of serotonergic neurons within the medium raphe nuclei (mRN) was not changed in different transgenic mouse models of PD, the innervation of the hippocampal formation (stained for serotonin transporter, SERT) and the glomerular layer of the olfactory bulb was diminished together with depressive symptoms and impaired olfaction, respectively.

In order to get a more precise insight into the temporal pattern of degenerative events, several animal as well as cellular models are relevant:

Numerous transgenic animal models for PD have been generated, mostly carrying mutations in genes associated with monogenic PD such as alpha-synuclein, leucine rich repeat kinase 2 (LRRK2), Parkin, PTEN-induced putative kinase 1 (PINK1), and protein deglycase DJ-1 (reviewed by Lee et al., 2012). Longitudinal studies provide important information about the temporal progression of neurodegenerative events within the dopaminergic system (**Figure 1**).

Chung et al. (2009) injected adeno-associated virus (AAV) 2 encoding for human A53T alpha-synuclein into the SN of rats. After 4 weeks, dopaminergic axons within the striatum were dystrophic and swollen, and levels of dynein, mediating retrograde axonal transport, were significantly decreased. Eight weeks after AAV injection, several microtubule based motor proteins required for anterograde transport were reduced in the striatum. Since these findings were not observed in the SN, an initial impairment of dopaminergic axons is implied. BAC alpha-synuclein transgenic mice, expressing human alpha-synuclein had a reduced striatal dopamine release without a loss of dopaminergic SN neurons at 3 months of age (Janezic et al., 2013). In contrast, the number of dopaminergic SN neurons was significantly reduced in aged (18-month-old) mice accompanied by a motor phenotype. This temporal pattern of structural and functional measures indicated an early axonal phenotype prior to the loss of SN perikarya. Using another BAC alpha-synuclein construct, an increased DAT level in whole brain lysates without reduction of dopaminergic SN neurons was observed in 24-month-old mice (Yamakado et al., 2012). BAC transgenic rats overexpressing full-length human alpha-synuclein show a severe motor impairment at 12 months of age with severe striatal dopamine depletion. Importantly, TH^+^ striatal fibers and TH^+^ SN neurons were reduced in 18-month-old rat, but not in 3-month-old rat (Nuber et al., 2013).

While there was already some evidence for the onset of PD pathology within projection areas of dopaminergic neurons, recent animal studies revealed a similar temporal pattern of events within the serotonergic system of murine PD models (**Figure 1**).

Aforementioned BAC transgenic rats (published by Nuber et al., 2013) additionally display severe impairment of hippocampal neurogenesis, a feature strongly associated with early behavioral phenotypes like depression and anxiety at 4 months of age, even prior to the onset of a motor phenotype (Kohl et al., 2016). Impaired hippocampal neurogenesis was accompanied by a profoundly reduced serotonergic innervation of the hippocampal dentate gyrus (DG; Kohl et al., 2016). Furthermore, 5-HT receptor expression was altered and 5-HT levels were significantly reduced in the hippocampal DG and CA3 region of transgenic animals, strongly indicating an early dysfunction of serotonergic terminals. The number of serotonergic neurons in the RN, however, remained unaltered. This temporal pattern of structural changes and the functional phenotype in the BAC transgenic rat model demonstrates that an early axonal phenotype preceding the loss of perikarya is also present in the serotonergic system in PD. Further evidence for an early axonal phenotype within the serotonergic system derives from the A53T alpha-synuclein mouse model (A53T mice) of PD. At 52 weeks of age, human synuclein was expressed in serotonergic RN neurons and their projections within the hippocampal formation (Deusser et al., 2015). While the number of serotonergic neurons was preserved in A53T mice, the density of serotonergic fibers within the dorsal DG was severely reduced. Furthermore, viral injections allowing the overexpression of alpha-synuclein selectively in serotonergic neurons resulted in a progressive degeneration of serotonergic axon terminals in the hippocampus, while the number of serotonergic RN neurons remained unaltered (Wan et al., 2016). Mutations in PINK1 are linked to monogenic forms of PD, as well. PINK1 deficient mice showed no motor phenotypes, but olfactory deficits at 19 months of age (Glasl et al., 2012). Olfactory dysfunction is a very common NMS in PD. Interestingly, serotonergic innervation was significantly diminished in the glomerular layer of the olfactory bulb, while again there was no reduction in the numbers of serotonergic RN neurons. This study also strongly indicates an early loss of serotonergic terminals in a second target region besides the hippocampal formation associated with NMS in PD.

Besides transgenic PD models acute neurotoxic models using toxins such as 1-methyl-4-phenyl-1,2,3,6-tetrahydropyridine (MPTP) or 6-hydroxydopamine (6-OHDA) specifically target dopaminergic SN neurons (reviewed by Blandini and Armentero, 2012). 6-OHDA is commonly injected directly into the striatum or the medial forebrain bundle, thereby directly damaging dopaminergic terminals. Few studies applied unilateral intranigral injections and revealed a severe loss of TH^+^ neurons associated with a severe reduction of dopaminergic fibers (Moses et al., 2008; Grealish et al., 2010). For systemic application of MPTP which specifically diminishes dopaminergic neurons a severe loss of TH^+^ striatal fibers was observed, but the temporal pattern of these events was not analyzed in detail. One day after MPTP treatment Serra et al. detected a 60% reduction of striatal dopaminergic fibers without significant increase in apoptotic TUNEL positive cells within the SN (Serra et al., 2002).

## Neurite Degeneration in Human Stem Cell Models of PD

The opportunity to model PD by reprogramming patient derived cells into induced pluripotent stem cells (iPSC) and differentiating them into neurons recently advanced the availability of human cellular models of PD. Human dopaminergic neurons of sporadic and LRRK2 PD patients (carrying the *G2019S* mutation) showed markedly less neurites per dopaminergic neuron and shorter neurites in both patient cohorts after 75 days of neuronal differentiation. The number of caspase3 positive cells, however, was also significantly increased in patient derived neurons pointing toward higher rates of cell death in these cultures. The neuritic phenotype appeared rather simultaneously to the degeneration of the dopaminergic neurons in this iPSC derived PD model. Using another approach to generate dopaminergic neurons from LRRK2 patient derived iPSC carrying the identical G2019S mutation, Borgs et al. (2016) detected a different phenotype. LRRK2 patient derived neurons that were generated from neural progenitor cells using small molecules showed a reduced total neurite length and increased neurite branching while the number of TH positive neurons was not altered after 35 days of differentiation.

## Concluding Remarks/ Perspectives

Enormous progress in imaging techniques, a large variety of animal models and the availability of PD patient iPSC derived neurons helped to significantly improve our knowledge of the temporal pattern of pathomechanisms underlying PD. Given the early appearance of NMS in PD and their tremendous impact on the patients’ quality of life it is important to compare the timeline of pathogenic events within the serotonergic system with those more soundly explored in the dopaminergic system. One important recent milestone in PD research represents the appreciation that the onset of the disease may take place at the synaptic site and within the axonal compartment. Another milestone in PD research is the focus on other neurotransmitter systems besides dopamine, since especially early phenotypes of PD are strongly associated with e.g., alterations of the serotonergic system. One possible mechanism underlying axonal degeneration in PD may be the impairment of axonal transport due to protein aggregation, cytoskeleton instability, or motor protein dysregulation (Lee et al., 2006; Esteves et al., 2010; Volpicelli-Daley et al., 2014). Whether protein aggregation within the axon, motor protein dysfunction, or disturbance of motor protein binding to microtubules initiates the impairment of axonal transport remains to be elucidated. Another initial mechanism may be the reorganization of the synapse including altered availability of neurotransmitter receptors and transporters. Focusing on the mechanisms of axonal degeneration both in the dopaminergic and the serotonergic system or potentially even inducing axonal regeneration may be a very promising strategy to intervene at a very early stage with disease progression.

## Author Contributions

JG, JW and ZK wrote the manuscript and designed figures/tables.

## Conflict of Interest Statement

The authors declare that the research was conducted in the absence of any commercial or financial relationships that could be construed as a potential conflict of interest.
